# Factors that influence the willingness of young adults in Dar es Salaam, Tanzania, to participate in phase I/II HIV vaccine trials

**DOI:** 10.3402/gha.v7.22853

**Published:** 2014-02-25

**Authors:** Theodora Mbunda, Muhammad Bakari, Edith A. M. Tarimo, Eric Sandstrom, Asli Kulane

**Affiliations:** 1 Department of Internal Medicine, School of Medicine, Muhimbili University of Health and Allied Sciences, Dar es Salaam, Tanzania; 2 Global Health, Department of Public Health Sciences, Karolinska Institutet, Stockholm, Sweden; 3 Department of Nursing Management, School of Nursing, Muhimbili University of Health and Allied Sciences, Dar es Salaam, Tanzania; 4 Department of Clinical Science and Education, Sodersjukhuset, Karolinska Institutet, Stockholm, Sweden

**Keywords:** HIV vaccine trial, young adults, willingness, Tanzania

## Abstract

**Background:**

HIV/AIDS continues to destroy the lives of young people especially in low-income countries. The inclusion of youths in HIV vaccine trials is of utmost importance in obtaining an effective vaccine that is acceptable to them.

**Objective:**

To characterize the willingness of young adults in Tanzania to participate in an HIV vaccine trial and the factors that influence this willingness.

**Design:**

Four hundred and fifty young adults who visited a youth-friendly Infectious Diseases Clinic (IDC) from February 2012 to September 2012 completed a self-administered questionnaire concerning sociodemographic information, their knowledge about and perception of HIV vaccine studies, and the availability of social support.

**Results:**

Of our participants, 50.6% expressed willingness to participate in HIV vaccine trials, and this willingness was positively correlated with having some knowledge about HIV vaccine studies (AOR, 2.2; 95% CI: 1.4–3.4), a positive perception toward such studies (AOR, 2.3; 95% CI: 1.5–3.6), having a relationship with someone who could help them make a decision (AOR, 2.5; 95% CI: 1.3–4.9), and age at the time of sexual debut (AOR, 2.6; 95% CI 1.0–6.7) for 15- to 19-year-olds and (AOR, 2.7; 95% CI 1.0–7.1) for older participants.

**Conclusions:**

The participants exhibited a moderate willingness to participate in HIV vaccine trials, which was associated with a positive perception of and some knowledge about such trials, having a relationship with someone who might influence their decision as well as age at time of sexual debut. More efforts should be made to inform the youths about specific HIV vaccine trials and related matters, as well as to engage significant others in the decision-making process.

With 34 million people living with HIV at the end of 2011, HIV/AIDS remains one of the most challenging diseases worldwide. With 69% of the population living with HIV, Sub-Saharan Africa is most severely affected (1), and among the countries in this region, Tanzania is one of the most seriously affected by the HIV/AIDS epidemic. Here, 5% of 15- to 49-year-olds (2) are infected, and the estimated prevalence in 2012 of 3.39 and 1.39% in women and men aged 15–24, respectively (3), indicates significant transmission between youths. The prevalence of HIV varies significantly between the different regions of Tanzania, the highest being in Njombe (15%) and the lowest in Pemba, 0.3%. In addition, this infection is more prevalent in urban (7%) than rural areas (5%) (2).

The primary route for transmission of HIV in Tanzania is unprotected heterosexual intercourse. Multiple concurrent partnerships, early marriage, inequitable gender norms, and gender-based violence contribute to spread of this virus (4). The disproportionate number of infected Tanzanian youths is due to sexual debut at an early age, infrequent use of condoms, and multiple concurrent sexual partners (2).

Currently, the national efforts focus on expanding and improving the quality of the prevention, care, treatment, and support for HIV/AIDS as well as mitigating the various socioeconomic consequences of the epidemic (5). By the end of 2012, approximately 740,040 people, accounting for 31% of the number estimated to be infected, were enrolled in care and treatment centers, In this same period, 51.9% of those enrolled into care had started anti-retroviral treatment (ART) (3).

Around the world, various measures not involving vaccination such as male circumcision and preexposure prophylaxis trials have been tried in an effort to revert new HIV infections. For instance, pre-exposure of uninfected 18- to 44-year-old women in KwaZulu-Natal, South Africa, to pericoital use of 1% tenofovir gel reduced the risk of HIV infection by 39%. Notably, the incidence of HIV among those receiving the placebo was 9.1 per 100 women-years, which is extraordinarily high (6).

In another investigation carried out in South Africa, Uganda, and Zimbabwe, daily treatment by oral Truvada or tenofovir vaginal gel was ineffective in preventing HIV, although there was increased HIV infection among young single women who used these products less frequently (7). Likewise, the Pre-exposure Prophylaxis Trial for HIV Prevention among African women (FEM-PrEP), involving high-risk uninfected women in Kenya, South Africa, and Tanzania, was stopped when equal numbers of infections were found in those administered Truvada or a placebo once each day (8).

Despite current and previous preventive efforts, new cases of HIV infection remain a problem in Tanzania, particularly among youths aged 15–24, who account for half of the new infections. Thus, the development of an effective HIV vaccine and the involvement of youths in testing such a vaccine is urgently needed for curbing the epidemic both in Tanzania and elsewhere. It is essential to evaluate the willingness of young people to participate in such trials, as well as factors that influence the willingness.

In the United States, 86% of the women enrolled reported that they were definitely or probably willing to participate in a trial of HIV vaccine, and this willingness to participate could be predicted from their own perceived personal benefit (9). In India, willingness to participate among men having sex with men is embedded in social, community, and familial spheres of influence. In this case, barriers to participation included fear of losing job opportunities, the stigma associated with vaccine induce seropositivity, as well as apprehension about side effects such as impotence (10). In South Africa, among 52.5% of the youths who indicated they were willing to participate, this willingness was motivated by receiving information about current HIV research, doing something to honor individuals infected with HIV or who had died of AIDS, and being offered cost-free counseling and testing (11). In Uganda, however, the willingness of people living in fishing communities to participate dropped from 99.4 to 90.6% when the trial required delaying pregnancy or drawing large amounts of blood (12).

In Tanzania, the willingness of adult police officers to volunteer for an HIV vaccine trial was influenced positively by the intention to tell significant others, a belief that the trial would be effective, the ability to make a personal decision, and the expectation of obtaining protection against HIV infection (13); and it was influenced negatively by personal fears, as well as the attitudes of significant others, including sexual partners, friends, family members, and relatives (14). Clearly, a willingness to participate in a vaccine trial is influenced not only by individual factors but also at the community level.

Since 2007, Tanzania has been involved in phase I/II HIV vaccine trials focusing on assessing the safety and immunogenicity of a candidate vaccine administered in various dosages and different routes (15). However, little is known about the HIV vaccine trial here, and even less is known about the willingness of young adults to participate in such a trial. Therefore, our present goal was to assess this willingness and the factors that influence it, with the aim of developing strategies to improve participation.

## Methods

### Settings

This study was conducted at the Infectious Disease Clinic (IDC) in Dar es Salaam, a government facility located in the Ilala Municipality that provides youth-friendly health care services such as screening and treatment of sexually transmitted infections, contraceptives, counseling, testing, care and treatment for HIV, as well as general information about adolescent sexual and reproductive health. In 2012, a total of 4,379 individuals, 72% of whom were adults and the remainder youths, aged 12–25 years, visited the IDC. Of the 1,237 young visitors, 816 were newly registered and 75% were females. More than 50% of those who registered for the first time came for HIV counseling and testing, with the next most common reason being STI screening and treatment.

### Study population

All individuals aged 18–25 years who visited the IDC from February to September 2012 were invited to participate. Of the 548 youths approached, 98 (18%) did not accept this invitation, primarily because of time constraints. The actual participants were then systematically selected by including every other respondent. For those who declined, their replacements were selected in the same manner until we reached the required sample size. Each participant filled out a self-administered questionnaire, written in Kiswahili, with minimal supervision from research assistants, who checked for completeness before double-entry of data into the computer. The participants were offered soft drinks at the clinic, and compensated for their transport costs in Tanzanian shillings (equivalent to 4 US dollars).

#### Ethical issues

The Institutional Review Board at Muhimbili University of Health and Allied Sciences (MUHAS) evaluated the study proposal and then granted an ethical approval (MU/DRP/AEC/Vol.XIV/33). All participants read and signed consent forms before joining the study.

### Data collection

The questionnaire gathered demographic information and information concerning knowledge about and perception of HIV vaccine studies, as well as about social support and sexual behavior.

### Data analysis

The frequency distributions of all data sets were determined. Bivariate analyses using the Chi-square test were performed to examine the associations between willingness to participate with and sociodemographic characteristics, perceptions, and knowledge concerning HIV vaccine studies, sexual behavior, and social support. Logistic regression models were employed to control for cofounders as follows: all bivariate analyses of willingness to participate with a *p*-value of <0.2 were included in multivariate logistic regression analyses and were considered to be statistically significant if the resulting *p*-value was <0.05 (16). The Hosmer–Lemeshow goodness of fit test was applied to assess the logistic regression model, and a final *p*-value of >0.05 taken as indicating good fit. The frequency of missing data was 3.3%. All statistical analyses were two-sided and performed using Stata Version 12.1 for Windows (StataCorp 1985–2011, College Station, Texas).

### Assessment of willingness to participate in an HIV vaccine study

The proportion of those willing to participate was determined based on a Yes or No response, and the association with demographic characteristics, knowledge, attitudes, and perceptions concerning HIV vaccine studies, social support, and sexual behavior was determined using binary logistic regression models as described above (yielding odds ratios (OR), *p*-values and 95% confidence intervals). Moreover, reasons of unwillingness to participate were explored through open-ended questions. The responses were grouped into categories consisting of concerns about safety, side effects, fear of HIV infection, and misconceptions as to who should use the vaccine. The frequencies of the responses were counted manually.

### Assessment of sexual behavior

The participants were asked if they ever had sexual intercourse, their age at coitarche, if they had had sexual intercourse during the past 6 months, if a condom was used during their latest sexual encounter, and if they had ever been raped. Bivariate associations between gender and sexual behavior were examined using the Chi-square test, where *p*<0.05 was considered to be statistically significant.

### Knowledge concerning HIV vaccine studies

Six questions assessed knowledge concerning the concept of placebo versus vaccine (one item), infectivity (three items), safety (one item), and protection afforded by (one item) the HIV vaccines. The questions were formulated by us, and they have not been validated by any standardized procedure. The Cronbach alpha coefficient was 0.6 and this index was included in the analysis despite its low internal reliability. Responses were scored as 1 when correct and 0 when incorrect or ‘I do not know’. Of the total possible score of 6, the mean was 2.1. Responses were categorized as ‘some knowledge’ scores at or above the mean and ‘low knowledge’ less than the mean.

### Perceptions of HIV vaccine studies

Eight statements were used to assess perceptions of HIV vaccine studies resulting in the Cronbach alpha coefficient of 0.64. All of these statements were Likert type with the following responses: strongly disagree, disagree, agree and strongly agree, scored as 1–4 respectively. With negatively phrased statements, these alternatives were listed as indicated above, whereas the order was reversed with positively phrased statements. The total score for each of the eight statements was determined for each participant; the median was 22. Any score ≥22 was considered as positive, and any score <22 was considered negative.

## Results

### Sociodemographic characteristics

Of the 450 youths who participated, 229 (51%) were women (Table 1). The demographic characteristics of the men and women were remarkably similar. Overall, the mean (SD) age was 21 (2.1) years, most (89%) participants were single, and 87.8% reported having no children (15% of women and 5% of men reporting having at least one child). The majority (80.4%) had a secondary or higher level of education. The educational level of 51.5% of the fathers/male guardians was secondary or higher, while the corresponding value for mothers/female guardians was 40.5%. Most (86.4%) reported having a significant other who might influence their decision.

**Table 1:** Sociodemographic characteristics of the participants

Variable	Men, *n* (%)	Women, *n* (%)	Combined, *n* (%)
Mean age (SD)			20.9 (2.1)
Marital status			
Single	202 (91.4)	197 (86.4)	399 (89.0)
Married/cohabiting	19 (8.6)	31 (13.6)	50 (11.0)
Participant's level of education			
Primary	42 (19.0)	42 (18.3)	84 (18.7)
Secondary or higher	275 (89.2)	187 (81.7)	362 (80.4)
No formal education	4 (1.8)	0	4 (0.9)
Father's/male guardian's level of education			
Primary	47 (21.4)	44 (19.2)	91 (20.3)
Secondary or higher	106 (48.2)	125 (54.6)	231 (51.5)
No formal education	9 (4.1)	8 (3.5)	17 (3.8)
‘I do not know’	58 (26.4)	52 (22.7)	110 (24.5)
Mother's/female guardian's level of education			
Primary	65 (29.4)	71 (31.0)	136 (30.2)
Secondary or higher	86 (38.9)	96 (41.9)	182 (40.5)
No formal education	12 (5.4)	9 (3.9)	21 (4.7)
‘I do not know’	58 (26.2)	53 (23.1)	111 (24.7)
Number of children			
None	200 (90.5)	195 (85.2)	395 (87.8)
>1	21 (8.5)	34 (14.9)	55 (12.2)
Availability of social support			
Yes	184 (83.3)	205 (89.5)	389 (86.4)
No	37 (16.7)	24 (10.5)	61 (13.6)

### Willingness to participate in HIV vaccine trials

Overall, 227 of the 450 participants (50.6%, 95% CI 46–55%) expressed a willingness to participate in HIV vaccine trials. The reasons given for unwillingness included concerns about safety (25%), fear of getting HIV infection from the vaccine itself (15%), and side effects (5%). Others believed that vaccines should not be tested on humans but animals instead. However, when they were asked if they would use an effective HIV/AIDS vaccine if available, 84.9% of 450 answered yes.

### Sexual behavior

As documented in Table 2, responses of women and men concerning sexual behavior were similar. Notably, most men (86.4%) and women (80.8%) reported being sexually active, with a mean age of 16.7 years at the time of sexual debut. The mean number of sexual partners was 2.8. Of 376, 77% reported having engaged in sexual activities during the past 6 months. Furthermore, the use of condoms in connection with the latest sexual activity was moderate, that is, 50.8 and 58.7% for women and men, respectively, *p*=0.13. Approximately 11.1% of 369 participants reported having been raped (70.7% females, 29.3% males, *p*=0.003).

**Table 2:** Sexual behavior reported by the participants

Variable	Men, *n* (%)	Women, *n* (%)	Combined, *n* (%)
Ever had sex	191 (86.4)	185 (80.8)	376 (83.6)
Mean number of lifetime partners (SD)			2.8 (2.7)
Age at coitarche			
<15 years	18 (12.7)	16 (10.7)	34 (4.8)
15–19 years	117 (82.4)	122 (81.9)	239 (89)
>20 years	7 (4.9)	11 (7.4)	18 (6.2)
Mean age at coitarche (+SD)			16.7 (2)
Sexual intercourse during the past 6 months	149 (78.0)	141 (76.2)	290 (77.1)
Condom use in connection with the latest sexual encounter	111 (58.7)	93 (50.8)	204 (54.8)
Ever been raped	12 (6.3)	29 (16.2)	41 (11.1)

### Knowledge about and perceptions of HIV vaccine studies

The 35.8% demonstrated some knowledge about HIV vaccine studies (37.6% for women and 33.9% for men, *p*=0.42) and 60.2% a positive perception (67.4% of the men, and 53.3% of women, *p*=0.002). Eighty three percent agreed that participation in HIV vaccine research was one of the strategies for reducing the worldwide threat from AIDS (Table 3).

**Table 3:** The knowledge about, and perception of, HIV vaccine studies by our participants

Variable	Men, *n* (%)	Women, *n* (%)	Combined, *n* (%)
Level of knowledge			
Some	75 (33.9)	86 (37.6)	161 (35.8)
Low	146 (66.1)	143 (62.5)	289 (64.2)
Perception			
Positive	149 (67.4)	122 (53.3)	271 (60.2)
Negative	72 (32.6)	107 (46.7)	179 (39.8)

### Factors that influence willingness to participate in an HIV vaccine trial

Application of the unadjusted logistic regression model at the univariate level, revealed no association between willingness to participate and age, gender, marital status, mother's/guardian's level of education, father's/guardian's level of education, having had sex, sexual activity during the past 6 months, having been raped, or the use of condoms in connection with the latest sexual encounter. However, this willingness was associated with the participants’ level of education (OR for secondary education or above=2.1 (95% CI 1.29–3.46)); age at the time of sexual debut (OR for the age groups 15–19 and older=3.4 (95% CI 1.46–7.77) and 3.6 (95% CI 1.55–8.46), respectively), some knowledge about HIV vaccine studies (OR 1.8, 95% CI 1.24–2.73); a positive perception of such studies (OR 2.2, 95% CI 1.5–3.2); and the availability of social support in decision-making (OR 2.2, 95% CI 1.15–4.1). The factor ‘not having children’ was marginally correlated (OR 1.77, CI 0.99–3.16).

After application of the adjusted logistic regression model, at the multivariate level, the factors that were still significantly associated with willingness to participate were some knowledge about HIV vaccine studies (AOR 2.1, (95% CI 1.5–3.4)); marginally correlated with age at the time of sexual debut (AOR for age groups 15–19 years and the older participants=2.6 (95% CI 1.0–6.7) and 2.7 (95% CI 1.0–7.1) respectively), a positive perception of such studies (AOR 2.3, (95% CI 1.5–3.6)); and availability of social support in connection with decision-making (AOR 2.5 (95% CI 1.3–4.9)) (Table 4).

**Table 4:** Factors that influence willingness to participate in HIV vaccine studies as assessed by univariate and multivariate analysis

	Willingness to participate (column %)					
	
	Univariate analysis				Multivariate analysis	
		
Variable	Total number	*n* (%)	Crude OR (95% CI)	*p*	AOR (95% CI)	*p*
Mean age (SD)	450	20.9 (2.1)	1.04 (0.95–1.14)	0.4		
Sex						
Male	220	109 (49.5)	0.99 (0.46–1.34)	0.9		
Female	229	118 (51.5)	1			
Marital status						
Not married	398	206 (51.8)	0.62 (0.34–1.13)	0.1	0.9 (0.4–1.9)	0.7
Married	50	20 (40)	1		1	
Number of children						
No child	394	206 (52.3)	1.77 (0.99–3.16)	0.05	1.4 (0.6–3.0)	0.5
Having one or more children	55	21 (38.2)	1		1	
Participant's level of education						
Secondary or higher	361	195 (54.0)	2.11 (1.29–3.46)	0.003	1.6 (0.9–2.8)	0.1
Primary	84	30 (35.7)	1		1	
Father's/guardian's level of education						
No formal education	17	8 (47.1)	1			
Primary	91	36 (39.6)	0.74 (0.26–2.09)	0.6	0.7 (0.2–2.2)	0.5
Secondary or higher	231	129 (55.8)	1.42 (0.53–3.82)	0.5	1.2 (0.4–3.7)	0.8
I do not know	109	54 (49.5)	1.10 (0.40–3.07)	0.8	1.0 (0.3–3.2)	1
Mother's/guardian's level of education						
No formal education	21	9 (43.9)	1			
Primary	135	63 (46.7)	1.17 (0.46–2.95)	0.7		
Secondary or higher	182	101 (55.5)	1.66 (0.67–4.14)	0.3		
I do not know	111	54 (48.7)	1.26 (0.32–1.78)	0.6		
Level of knowledge about HIV vaccine studies						
Some knowledge	161	97 (60.3)	1.84 (1.24–2.73)	0.002	2.2 (1.5–3.4)	0.000
Low knowledge	288	130 (45.1)	1		1	
Perception of HIV vaccine studies						
Positive	271	157 (69.2)	2.16 (1.47–3.18)	0.000	2.3 (1.5–3.6)	0.00
Negative	179	70 (30.8)	1		1	
Ever had sexual intercourse						
Yes	375	189 (50.4)	0.96 (0.58–1.59)	0.9		
No	74	38 (51.3)	1			
Age at the first sexual encounter						
<15 years	33	8 (24.2)	1			
15–19	239	124 (51.9)	3.37 (1.46–7.77)	0.004	2.6 (1.0–6.7)	0.05
>19	177	95 (53.7)	3.62 (1.55–8.46)	0.003	2.7 (1.0–7.1)	0.05
Condom use in the last sexual encounter						
Yes	203	97 (47.8)	0.79 (0.5–1.19)	0.3		
No	168	90 (53.6)	1			
Sexual intercourse in the past 6 months						
Yes	289	143 (49.5)	0.85 (0.53–1.38)	0.5		
No	86	46 (53.5)	1			
Rape						
Yes	41	18 (43.9)	0.75 (0.39–1.44)	0.4		
No	327	167 (51)	1			
Having someone important who may influence decision						
Yes	388	205 (52.8)	2.17 (1.15–4.1)	0.02	2.5 (1.3–4.7)	0.01
No	47	16 (34)	1		1	

## Discussion

Here we found that no more than 50.6% of young adults in Dar es Salaam are willing to participate in HIV vaccine trials despite the fact that this age group runs the highest risk for new HIV infection and thus has most to benefit from an efficacious vaccine. This finding is relatively similar to the 40% of youths in South Africa who reported willingness to participate (17) but lower than 82 and 95% observed in South India and Uganda, respectively (18, 19). Some of the reasons our participants gave for unwillingness included a fear of getting HIV infection from the vaccine itself, concerns about safety, and side effects. In Uganda, the participants were concerned about the safety of candidate vaccines, large blood draws, and time for clinic visits (19). These findings reflect at least to some degree, misconceptions about vaccine trials, which could be, in part due to the fact that participation of Tanzanian youths in HIV vaccine trial is relatively new. Previously, most clinical trials concerning infectious diseases such as polio and tuberculosis have been conducted in high-income countries, as were earlier HIV vaccine trials most in African countries, so that Tanzania was not involved. To obtain the large number of participants required for trials of efficacy, more effort must be put into informing youths about the importance of conducting and participating in such trials, and alleviating unfounded fears and anxieties. At the same time, 85% of our participants were willing to use an effective HIV vaccine if one becomes available, a finding similar with adult police officers in Tanzania (13). This illustrates that while the young people may not necessarily want to be trial participants, they would not hesitate to use an effective vaccine because they do not want to become infected with HIV.

We found that as many as 64.2% of our participants demonstrated little knowledge of HIV vaccine studies which may be due to inadequate dissemination of information particularly to the young, leading to misconceptions, anxieties, worries, and incorrect interpretation of such information. This observation was similar to the report from Uganda that adolescents there have poorer knowledge of HIV and HIV vaccines than adults (20), whereas in India subjects reported difficulties in understanding and accepting the concepts of HIV vaccine trials, including the use of placebo and double blind, and felt that ordinary people would find it very difficult to understand these concepts (10). Such observations emphasize the importance of empowering the youth with comprehensive information designed to satisfy their specific needs before they enroll in a trial. However, it has been argued that general education may not adequately address misconceptions about trials, and different forms of educational intervention need to be more thoroughly evaluated in this context (21).

Our current findings demonstrated that a willingness to participate was significantly correlated to having some knowledge about the HIV vaccine, which may allow the youths to feel more confident about participating if they know more issues specific to the HIV vaccine trial. This observation is in good agreement with findings from a study in South Africa (17), but in contrast to a study in the United States in which young people were less willing to participate in a potential HIV vaccine trial after receiving comprehensive information about the vaccine (22). It is important that young people have shown willingness to participate in vaccine trials, however it has been discussed that researchers need to optimize understanding of the scientific and technical details of such trials, as well as to examine the meanings of commonly used terms (23). All in all, given such a predicament observed between providing comprehensive knowledge and willingness to participate in a vaccine trial observed in earlier studies, strategies to address both aspects are to be taken on board when conducting clinical trials, as this is ethically important when dealing with human subjects.

In our present study, 60.2% of the participants demonstrated a positive perception of HIV vaccine studies and 83% agreed that participation in such research was one way to help reduce the worldwide threat of AIDS. Furthermore, willingness to participate was significantly correlated with such a positive perception. This indicates that young people know the gravity of HIV burden, recognizing that such a vaccine may be one of the ways to control this pandemic.

In addition, willingness to participate was also positively correlated with having someone close who might influence the decision-making process by giving advice; 53% of the respondents reported having such a relationship. This is of a particular interest since most young people appear to be more independent, wanting to get away from some customs, traditions, and norms, existing in many African societies, that they may regard as restrictive by not involving many people around them with regard to certain decisions. Indian men having sex with men, and still living with their parents reported that it was important for them to tell family members about the trial and to get their approval; while others thought about asking for support from their male partners. In this study, important individual concerns were embedded in broader familial, community, and structural domains (10). Similarly, willingness to participate among police officers in Tanzania was significantly correlated with the intention to tell significant others (13); while in Kenya female participants were more likely and even more expected than men to consult and seek permission from partners, family members, peers, and even research staff (24).

This underscores the significant influence of people close to the participants seen in many communities where numerous important decisions are usually not made by the youths alone, but rather together with those who surround them.

Moreover, the community influences on the decision to enroll and participate in HIV vaccine trials have received little attention (24), and Tanzania is no exception in this respect. It is therefore imperative to devise strategies for involving significant others in the process of recruiting young people into HIV vaccine trials.

One limitation of the current investigation is that information on willingness to participate in a hypothetical vaccine trial was examined, so that the findings might not accurately reflect the situation with a real trial. Moreover, self-reported sexual behavior might be biased by a reluctance to break societal norms. Furthermore, our research tool has not been validated in Dar es Salaam against other reliable research tools. The questions designed to assess perception and knowledge had low internal reliability and our findings should therefore be interpreted with caution, although we decided to include them because of their importance in this topic. Finally this study was performed at IDC, a youth clinic in Dar es Salaam, and the results may not be applicable to other youths who do not have access to a medical facility or choose not to visit the IDC.

## Conclusion and recommendations

The moderate willingness of the youth in Dar es Salaam to participate in an HIV vaccine trial was influenced positively by having some knowledge and positive perception of studies, as well as having someone important to discuss their decision with. To optimize the recruitment and retention of youths in HIV vaccine trials, they must be informed about concepts involved, employing approaches designed to facilitate their understanding, and address their concerns. Moreover, it is imperative that in this context, researchers and other stakeholders engage people who are important to the youths in the decision-making process.
